# A Chemiluminescent Protein Microarray Method for Determining the Seroglycoid Fucosylation Index

**DOI:** 10.1038/srep31132

**Published:** 2016-08-16

**Authors:** Aiying Zhang, Sven Skog, Shengqi Wang, Yang Ke, Yonghong Zhang, Kang Li, Ellen He, Ning Li

**Affiliations:** 1 Beijing Institute of Hepatology, Beijing YouAn Hospital, Capital Medical University, Beijing 100069, China; 2 Sino-Swed Molecular Bio-Medicine Research Institute, Shenzhen 518057, China; 3 Department of Biotechnology, Beijing Institute of Radiation Medicine, Beijing 100850, China; 4 Peking University Health Science Center, Beijing 100191, China; 5 Beijing YouAn Hospital, Capital Medical University, Beijing 100069, China

## Abstract

The *Lens culinaris* agglutinin-reactive fraction of AFP (AFP-L3) is widely used to screen for hepatocellular carcinoma (HCC) in Japan and China. We developed a chemiluminescent protein microarray for determining the AFP-L3/AFP index (the ratio of AFP-L3 to total AFP, AFP-L3%) by fixing AFP-specific antibodies and *Lens culinaris* lectin on aldehyde-coated glass slides. Serum samples were tested for AFP using an enzyme-linked immunosorbent assay (ELISA) to validate the microarray. AFP-L3 was detected using Hotgen Biotech glycosyl capture spin column pretreatment technology and ELISA. When the AFP cut-off value was set to 20 ng/ml, the protein microarray displayed 89.74% sensitivity and 100% specificity for HCC diagnosis, and the ELISA displayed 87.17% sensitivity and 100% specificity. When the AFP-L3% cut-off value was set to 0.1, the protein microarray displayed 56.41% sensitivity and 100% specificity for HCC diagnosis, and the ELISA displayed 53.84% sensitivity and 100% specificity. The ROC curve for the HCC diagnosis showed that the AFP area under the ROC curve (AUC = 0.996; 95% CI: 0.986–1.005) was much higher than that of AFP-L3 (AUC = 0.857; 95% CI: 0.769–0.94) and AFP-L3% (AUC = 0.827; CI: 0.730–0.924). The microarray assay used in this study is a highly sensitive, accurate, and efficient assay for the determination of the AFP-L3%.

Alpha-fetoprotein (AFP) produced by primary hepatic cancer is different from that generated by hepatitis, hepatic cirrhosis, and other benign hepatic diseases with respect to the carbohydrate chain. Compared with AFP generated by benign hepatic diseases, AFP generated by hepatic cancer has a much higher fucosylation index. Fucose has the characteristic of binding to *Lens culinaris* agglutinin (LCA), which is isolated from *Lens culinaris* (lentil) seeds. It has two subunits and a molecular weight of 46 kDa, and it forms a complex with sucrose. LCA is a useful component in affinity chromatography columns for the separation of glycoconjugates. AFP can be categorized into AFP-L1, AFP-L2, and AFP-L3 according to the affinity of the fucose residues for LCA. AFP-L3 has a high binding affinity for lectin LCA. AFP-L1 is mainly produced in benign hepatic diseases, AFP-L2 is mainly produced by pregnant women, and AFP-L3 is mainly produced in hepatocellular carcinoma (HCC)^1^,^2^,^3^. In 2005, the Food and Drug Administration (FDA) of the United States of America (USA) approved the use AFP-L3 as a tumor marker for primary hepatic cancer. AFP-L3 has high specificity and sensitivity for early diagnosis, differential diagnosis, evaluations of therapeutic effects, and prognosis monitoring^4^,^5^,^6^.

Fucose is a methylated hexose that exists in carbohydrate chains of various glycoproteins in tissue and serum and is referred to as protein-bound fucose (P-bf). A fucose residue is present in the carbohydrate chain of AFP. This heteroplasmon is called fucosylated AFP (FucAFP), and its percentage of the total amount of AFP is called the fucosylation index (Fuol)^7^,^8^,^9^. The Fuol has important theoretical and clinical significance and can be used as an important indicator in hepatic cancer diagnostic and prognostic applications. AFP-L3 is an indicator of the biological behavior of HCC. The AFP-L3 levels correlate with patient survival and treatment. AFP-L3-positive HCC has the potential for rapid growth and early distant metastasis. Elevated AFP-L3 levels are considered to indicate treatment failure.

The conventional serum fucose protein separation method involves the crossed affinity immunoelectrophoresis technique^10^,^11^, affinity blotting^12^,^13^, affinity chromatography^14^,^15^, a “dual-site sandwich” enzyme- linked immunosorbent assay^16^, a LiBASys tester (Chuo-ku Japan), the μTASWako^®^ i30 detection system technology (Richmond, VA USA), and the Hotgen Biotech glycosyl capture spin column pretreatment technology (Beijing, China). Of these components, the phytolectin affinity immunoelectrophoresis technique and the μTASWako^®^ i30 detection system technology have sophisticated requirements and use expensive reagents, thus restricting their popularization and application. Moreover, the glycosyl capture spin column makes the procedures more complicated because the sample treatment and detection are separated.

In this study, a protein microarray method was developed to quantitatively detect AFP and/or FucAFP in biological samples and address the lack of quantitative detection technology for AFP and AFP-L3 in serum. The application of the protein microarray technique to AFP is not novel, but its application to AFP-L3 is novel. To our knowledge, a protein microarray method for detecting AFP-L3 has not been reported previously. This method is applicable both for the detection of AFP antigens in serum as well as for the general detection of other fucosylated proteins. Moreover, it has the advantages of being time-saving, inexpensive, accurate, and convenient.

## Results and Discussion

All serum samples, including those from subjects with HCC and healthy controls, were detected by the AFP-L3% protein microarray assay. The serum AFP and AFP-L3 levels were markedly higher in patients with HCC than in the healthy individuals. The current AFP detection level adopts 20 ng/ml as the boundary^17^,^18^, with healthy individuals having AFP values of less than 20 ng/ml. An AFP-L3% >10–15% is a positive indicator of HCC^17^.

**Figure 1:**
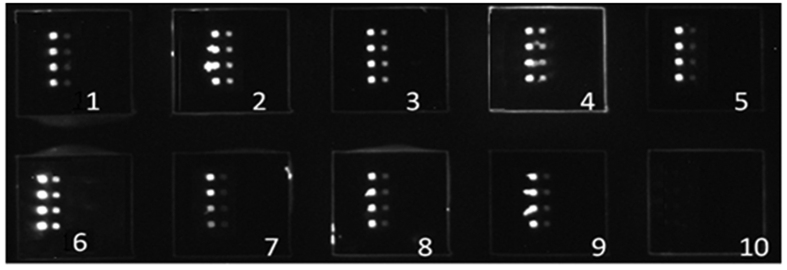
Scan chart of HCC serum and normal healthy serum samples detected by the AFP/LCA protein microarray assay. HCC serum samples, 1–9; normal healthy serum sample, 10.

Serum samples obtained from 39 healthy blood donors and 10 blank control solutions were tested to evaluate the detection specificity of the protein microarray. The scan charts of nine HCC serum samples and one normal healthy serum sample are shown in Fig. 1. Neither AFP levels greater than 20 ng/ml nor AFP-L3% greater than 10% was obtained from the healthy serum samples, which indicates that the false positive rate of the microarray presented here was very low or even zero. AFP levels less than 20 ng/ml but greater than 0 were detected in 2 of 39 healthy samples, and an AFP-L3% of less than 10% was detected in 1 of 39 healthy samples. Using the ELISA assay, AFP levels less than 20 ng/ml but greater than 0 were detected in 3 of 39 healthy samples, and an AFP-L3% of less than 10% was detected in 1 of 39 healthy samples. Neither AFP levels greater than 20 ng/ml nor an AFP-L3% greater than 10% was obtained from the healthy serum samples by ELISA.

The results of the protein microarray showed that AFP was detected in 37 of 39 HCC samples (94.87%). An AFP level greater than 20 ng/ml was observed in 35 of 39 HCC samples (89.74%). Both AFP and AFP-L3 were detected in 26 of 39 HCC samples. Neither AFP nor AFP-L3 was detected in 2 of 39 HCC samples. An AFP-L3% greater than 10% was observed in 22 of 26 HCC samples (84.61%), whereas an AFP-L3% less than 10% was observed in 4 of 26 samples. All the data are summarized in Table 1. The protein microarray assay exhibited a sensitivity of 89.74% and a specificity of 100% for detecting AFP and thus can reliably be used in clinical applications.

All the samples described above were also tested using the Hotgen Biotech glycosyl capture spin column pretreatment technology and ELISA assay at the clinical laboratory of Beijing YouAn Hospital. Previous data using Hotgen Biotech glycosyl capture spin column pretreatment and ELISA were compared with the protein microarray results from the present study to evaluate the microarray assay. AFP was detected in 37 of 39 HCC samples (94.87%). An AFP level greater than 20 ng/ml was observed in 34 of 39 HCC samples (87.17%). Both AFP and AFP-L3 were detected in 26 of 39 HCC samples. Neither AFP nor AFP-L3 was detected in 2 of 39 HCC samples. An AFP-L3% greater than 10% was observed in 21 of 26 HCC samples (80.76%), whereas an AFP-L3% less than 10% was observed in 5 of 26 samples (Table 1). There were no statistically significant differences between the ELISA and protein assays in either group (P > 0.05; χ2-test). There was a statistically significant difference in the protein microarray and ELISA measurements between the HCC patients and healthy controls (P < 0.01). When the AFP cut-off value was set to 20 ng/ml, the protein microarray displayed 89.74% sensitivity and 100% specificity for HCC diagnosis, whereas the ELISA displayed 87.17% sensitivity and 100% specificity. When the AFP-L3% cut-off value was set to 0.1, the protein microarray displayed 56.41% sensitivity and 100% specificity for HCC diagnosis, and the ELISA displayed 53.84% sensitivity and 100% specificity.

**Figure 2:**
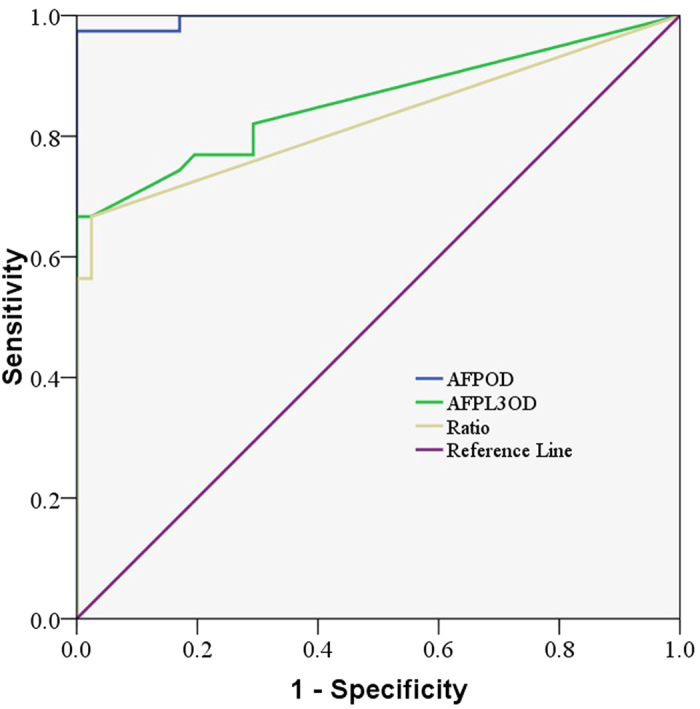
Receiver operating characteristic curve of the serum a-fetoprotein (AFP) and AFP-L3 levels from the protein microarray for the diagnosis of hepatocellular carcinoma in patients with hepatocellular carcinoma and healthy controls (n = 78). The area under the ROC curve (AUC) indicates the clinical usefulness of a tumor marker. Our results showed that the AFP area under the ROC curve (AUC = 0.996; 95% CI: 0.986–1.005) was much higher than that of AFP-L3 (AU = 0.857; 95% CI: 0.769–0.94) and AFP-L3% (AUC = 0.827; CI: 0.730–0.924).

The ROC curve for the diagnosis of HCC is shown in Fig. 2. The area under the ROC curve (AUC) indicates the clinical usefulness of a tumor marker. Our results showed that the AFP area under the ROC curve (AUC = 0.996; 95% CI: 0.986–1.005) was much higher than that of AFP-L3 (AUC = 0.857; 95% CI: 0.769–0.94) and AFP-L3% (AUC = 0.827; CI: 0.730–0.924). Our data showed that the elevated AFP-L3 levels positively correlated with the AFP levels (r = 0.974, P = 0.000). However, AFP-L3 did not correlate with the tumor stages, which may be due to the limited number of patients (Supplementary Information).

Here, we present a chemiluminescent protein chip for detecting the seroglycoid fucosylation index. The chemiluminescent protein chip is based on the antibody-antigen-antibody sandwich reaction and chemiluminescence principles^19^,^20^. The AFP-specific antibodies and LCA compounds were fixed on the surface of aldehyde-coated glass slides. The AFP-specific antibodies were used to bind all AFP (AFP-L1, AFP-L2 and AFP-L3) in serum, whereas the LCA was used to bind FucAFP (AFP-L3)^21^,^22^. Control spots were also included. Therefore, the total concentration of AFPs and the concentration of FucAFP in the serum could be simultaneously detected under identical conditions; thus, the seroglycoid fucosylation index could be accurately estimated. The anti-AFP antibody (0.5 mg/ml) and LCA (4 mg/ml) spotted on the aldehyde-coated slides were specifically bound to the AFP antigen and AFP-L3, respectively. AFP is a well known tumor-associated protein. Measurements of serum AFP levels are useful for detecting HCC. However, the correlation between serum AFP or AFP-L3 levels and the degree of histochemical AFP or AFP-L3 positivity has not been demonstrated^23^. The lack of signal in HCC patient (or healthy) samples reflects the low AFP and AFP-L3 concentrations. The reference range for total serum protein is typically 60–80 g/l. Concentrations below the reference range usually reflect a low albumin concentration, such as in liver disease or an acute infection. In this study, a retrospective study at Beijing Youan Hospital was performed among 43 HBV-infected patients, 79 patients with cirrhosis, and 189 patients with HCC (stage 0, 48; stage A, 47; stage B, 46; and stage C, 48). The AFP levels and total serum protein levels in the blood were measured in the 311 patients. Statistical analyses were conducted using SPSS version 17.0 (SPSS Inc., Chicago, IL, USA) for Windows. Our results showed that the serum AFP levels were negatively correlated with total serum protein levels (r = −0.113, P = 0.046).

Alpha-fetoprotein (AFP) is the main component of mammalian fetal serum. It is synthesized by the visceral endoderm of the yolk sac and by the fetal liver, and it is undetectable or only detected in trace amounts in adults. AFP-L3 is an isoform of alpha-fetoprotein (AFP), a substance typically used in the triple test during pregnancy and for screening patients with chronic liver disease for hepatocellular carcinoma (HCC). AFP is produced by the majority of hepatocellular carcinomas and is directly related to the severity of the disease^24^. In addition to the secretion of AFP, 90% of hepatocellular carcinoma cells express telomerase. It has also been reported that AFP can modulate the apoptotic signals induced by various cytotoxic factors by either promoting or inhibiting apoptosis^25^. A recent study showed that cytoplasmic AFP may function as a regulator of cell growth by interacting with PTEN (phosphatase and tensin homolog deleted on chromosome ten) and stimulating cell growth through the PI3K/AKT signaling pathway^26^.

In the first trimester of pregnancy, AFP levels were higher in women with the anti-HCV antibody than in women without it^27^. PLAP, CD117, AFP, and β-HCG are routine immunohistochemical markers for the diagnosis and differential diagnosis of malignant germ cell tumors^28^,^29^,^30^. AFP levels are also sometimes increased in patients with chronic viral hepatitis and cirrhosis who do not have HCC^31^,^32^,^33^,^34^. AFP and (or) AFP-L3 levels may be elevated in response to the above-mentioned diseases. Therefore, this assay can also be performed in pregnant women with acute or fulminant hepatitis, germ cell tumors, viral infections, and cirrhosis.

HRP is the most widely used catalyst in enzymatic reactions^35^,^36^. It is isolated from horseradish roots and contains fucose residues. If the samples were labeled with HRP, the fucose residues in the HRP would bind to the LCA, thus interfering with the test. Experiments performed in this study showed that an accurate fucosylation index cannot be obtained in this manner because the false positive rate is very high, and very high fucosylation index values can be obtained for normal serum. However, the protein microarray assay presented in this study can be used for qualitative detection and can also quantitatively determine the AFP and FucAFP levels based on the chemiluminescence intensity. The protein microarray presented here also provides a method for quantitatively determining the FucAFP levels. With the use of specific binding between antibodies and antigens and the specific binding between LCA and fucose combined with biotin-labeled AFP polyclonal antibodies, HRP-labeled avidin, and an HRP chemiluminescent substrate, the concentrations of AFP and fucose AFP-L3 in the sample can be obtained by inserting the acquired signal values from the scanner into a pre-established linear regression equation.

Although the ELISA can be combined with chemiluminescence, our protein microarray assay has many advantages compared with the ELISA method. 1) The serum AFP and FucAFP levels are detected under similar conditions to ensure that the detected fucosylation index is accurate and reliable. 2) Multiple samples can be analyzed simultaneously. Duplicate samples or samples taken at different time points can be analyzed to obtain dynamic values, or different samples can be analyzed, thus enabling high-throughput detection. Consequently, the detection cost can be reduced, and the detection efficiency can be improved. 3) The amounts of serum and antibodies required for the protein chip described here are substantially less than those required for other techniques: only 10 μl of original serum (or diluted) is needed, whereas 50 μl of original serum (or diluted) is required for the ELISA method. For antibody applications to the protein chips, 5 μl of serum can be applied to samples on at least 20 protein chips; thus, the amount of antibody required is substantially less than that of the ELISA method when 200 serum samples are analyzed, and the detection cost and expense can be greatly reduced. 4) The protein microarray was performed in 1.5–2 h in the present study (excluding the time required to manufacture the microarray), whereas the ELISA kit used in this experiment required >3.5 h. In conclusion, the chemiluminescence detection method, standard curve, and the protein chip technology ensure high sensitivity, accuracy, efficiency, and low cost when used for the quantitative analysis of AFP and AFP-L3 levels. The detection method reported here is a feasible, reliable, economical, simple, and time-saving method.

## Materials and Methods

### Collection of serum samples from patients and healthy controls

A total of 78 whole-blood samples from patients with HCC (n = 39) and healthy controls (n = 39) were collected at Beijing YouAn Hospital. Written informed consent was obtained from the participants prior to sample collection. The study protocol was approved by the Ethics Committee of Beijing YouAn Hospital. The methods were performed in accordance with the guidelines approved by the Ethics Committee of the Beijing YouAn Hospital, Beijing, China. The study recruited patients with HCC who underwent liver resection surgery or transcatheter arterial chemoembolization (TACE) and radiofrequency ablation (RFA) at the Department of Hepatobiliary Surgery, Beijing YouAn Hospital, Beijing, China. The patients were required to be positive for serum hepatitis B surface antigen and free from chronic hepatitis C infection. HCC diagnosis was histologically confirmed after surgical resection. The Barcelona Clinic Liver Cancer (BCLC) staging system is the most commonly used HCC management guideline. According to the BCLC staging system, HCC staging includes very early stage HCC or stage 0; early stage HCC; and stages A, B, C, and D (advanced stage). Not all HCC patients enrolled in these studies were categorized according to the BCLC staging system. Some patients were classified as stage 0, A, B, or C according to the BCLC staging system. The blood samples were centrifuged at 3,400 RPM/min for 10 min after clotting, and serum aliquots were stored at −70 °C. All diagnoses of HCC were histologically confirmed after surgical resection. Healthy volunteers with no diagnosis of gastrointestinal or hepatobiliary diseases or other types of diseases were recruited from the outpatient department and used as controls.

### Preparation of reagents and samples

The following reagents were used: Mouse monoclonal [AFP-11] to alpha 1 Fetoprotein antibody (Abcam Trading Company Ltd. Shanghai, China), Recombinant Human alpha 1 Fetoprotein (Abcam Trading Company Ltd. Shanghai, China), LCA (Sigma), aldehyde-coated glass chips (Shanghai Baiao, China), biotin-labeled anti-AFP antibody (Abcam Trading Company Ltd. Shanghai, China), streptavidin-horseradish peroxidase (Abcam Trading Company Ltd. Shanghai, China), RapidStep™ ECL (Merck KgaA, Darmstadt, Germany), and phosphate-buffered saline (PBS) buffer containing 0.05% Tween 20 (PBST). The chemiluminescence scanner used was developed by the laboratory of Professor Wang Sheng-Qi of the Academy of Military Medical Sciences. The biotin-labeled anti-AFP antibody was diluted 1:100 in PBST, pH 7.5. HRP-labeled streptavidin was diluted 1:100 in PBST. Serum was diluted 1:4 in PBST and 0.5% BSA, pH 7.5. After thawing on ice, all serum samples and antibody reagent solutions were stored for no longer than 7 days at 4 °C, although they are best within one day. In this study, the protein microarray and ELISA tests were performed within 24 h. The serum samples could be used to detect AFP and AFP-L3 after storage at 4 °C for 7 days. The results of the protein microarray using serum stored at 4 °C for 7 days were negligibly reduced compared to serum stored at 4 °C for one day.

**Figure 3:**
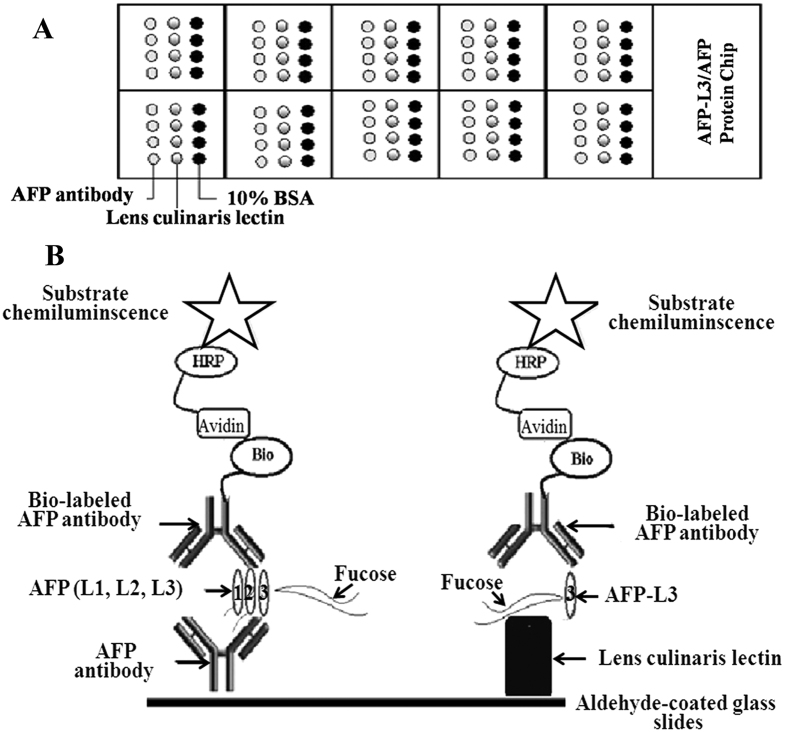
(**A**) The schematic of the AFP antibody and LCA applications on the protein chip. (**B**) The flow chart of the protein microarray chip AFP/LCA antibody sandwich method.

A hydrophobic paper containing 10 holes was stuck on the surface of an aldehyde-coated glass slide (chip) to form 10 reaction grids (see Fig. 3A). The anti-AFP antibody (0.5 mg/ml), LCA (4 mg/ml), and 10% bovine serum albumin (BSA) were spotted onto the aldehyde-coated slides in quadruplicate with a microarray spotter (Cartesian Technologies, USA) (Fig. 3A). BSA was used as a negative control. Following spotting, the slides were incubated for 24 h at 4 °C. The slides were blocked with 20 μl of blocking buffer (10% normal goat serum containing 0.1% NaN_3_) for 2 h at 37 °C and then washed four times with PBST. After drying, the microarray chips were stored at 4 °C until use.

### Running the protein microarrays

A sample of either 2.5 μl of serum or 10 μl of 1:4 diluted serum was added to each grid of the microarray and incubated for 30 min at 37 °C. Diluting the serum may decrease the ability of the assay to detect AFP and/or AFP-L3. When the serum AFP concentration was 1.25 ng/ml, AFP could be detected in the original serum using this assay. After further dilution, the AFP concentration was too low (lower than 1.25 ng/ml) to detect. The AFP antigen could not be detected in the diluted serum when the AFP level in the original serum was less than 1.25 ng/ml.

In this study, all serum samples were diluted 1:4 with PBST. When the AFP level in the original serum was greater than 5 ng/ml, the AFP antigen could be detected in the diluted serum (10 μl). The AFP-L1, -L2, and -L3 proteins in the serum were specifically bound to the mouse anti-AFP monoclonal antibodies, whereas the AFP-L3 protein in the serum was bound to LCA (Fig. 3B). We cannot exclude the possibility that AFP-L3 bound to the mouse anti-AFP monoclonal antibody. The chips were washed four times with PBST to eliminate non-specific binding. Subsequently, the PBS-diluted biotin-labeled rabbit anti-AFP polyclonal antibody was added, and the chips were incubated for 30 min at 37 °C. The chips were washed four times with PBST to eliminate non-specific binding. Subsequently, PBS-diluted streptavidin-HPR was added, and the chips were incubated for 30 min at 37 °C. The chips were again washed four times with PBST to eliminate non-specific binding. Subsequently, the chemiluminescent HRP substrate (RapidStep™ ECL) was added, and the chips were incubated for 30 min at 37 °C and then scanned with a chemiluminescent scanner developed by the laboratory of Professor Wang Sheng-Qi. This assay can reproduced using a commercially available scanner, ChemiDoc™ MP System (Bio-Rad, California 94547 USA). It was a full-feature instrument for protein microarray imaging designed for chemiluminescence detection applications. Its features are based on high-resolution, high-sensitivity CCD detection technology. The system is controlled by Image Lab software to optimize performance for the fast, integrated and automated image capture and analysis of various samples. The chemiluminescent pixel value (OD value) on a solid-phase carrier positively correlates with the amount of antigen in the sample. The chip-catching antibody (mouse anti-AFP monoclonal antibodies) and the antibody used for detection (biotin-labeled rabbit anti-AFP polyclonal antibody) were from different species of animals. Figure 3B shows a flow chart of the protein chip antibody sandwich method.

### Standard curves of AFP and AFP-L3

**Figure 4:**
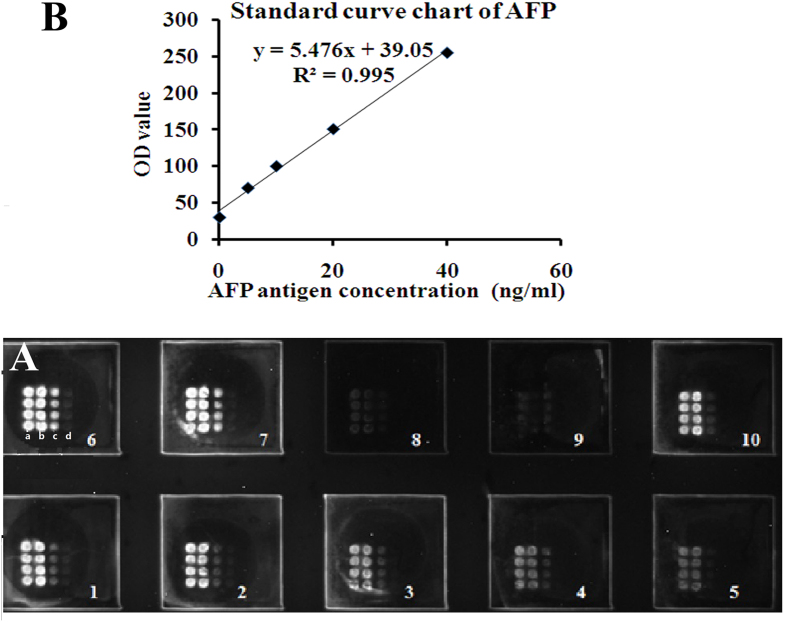
(**A**) AFP antigen levels detected by the AFP protein microarray. AFP antibodies were applied to the slides at different concentrations: a, 1 mg/ml; b, 0.5 mg/ml; c, 0.25 mg/ml; and d, 0.125 mg/ml. Different concentrations of the AFP antigen were used: 1.80 ng/ml, 2.40 ng/ml, 3.20 ng/ml, 4.10 ng/ml, and 5.5 ng/ml. Sera from HCC patients and healthy controls were also included: 6, HCC serum; 7, HCC serum; 8, blank control; 9, healthy serum; and 10, HCC serum. (**B**) The standard curve chart and the regression equation for determining the AFP levels using the AFP protein microarray assay.

The standard curve of AFP was created by applying different concentrations of AFP antigen (5, 10, 20, 40, and 80 ng/ml) to the microarray plate coated with the anti-AFP antibody, which was then incubated for 30 min at 37 °C (Fig. 4A,B). The anti-AFP capture antibody was applied to the slides at different concentrations: a, 1 mg/ml; b, 0.5 mg/ml; c, 0.25 mg/ml; and d, 0.125 mg/ml. AFP was detected as described above. The anti-AFP antibody (0.5 mg/ml) was used to design the standard curve by choosing the best concentration of anti-AFP antibody. Lower concentrations of AFP were undetectable when 0.25 mg/ml of anti-AFP antibody was applied to the slides. A higher concentration (1 mg/ml) of anti-AFP antibody was unsuitable for designing the standard curve because the chemiluminescent pixel values (OD value) were usually higher than the upper limit of the pixel analysis of this chip.

**Figure 5:**
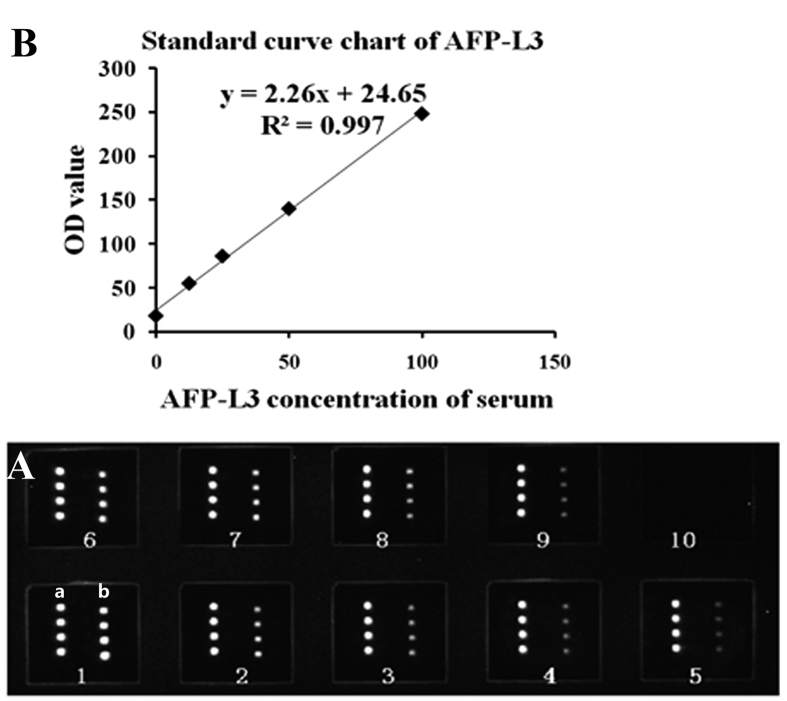
(**A**) Scan chart of AFP-L3 standard substances detected by the AFP/LCA-coated chips. AFP antibodies and LCA were applied to the slides. a: AFP antibody, 0.5 mg/ml; b: LCA, 4 mg/ml. Serum samples with different concentrations of AFP-L3 were used: (1–5) 100, 50, 25, 12.5, and 6.25 ng/ml; (6–9) 100, 50, 25, and 12.5 ng/ml; (10) blank control. **(B)** The standard curve chart and the regression equation of AFP-L3 determined using the AFP/LCA protein microarray assay.

Serum of a known AFP-L3 concentration was diluted in multiple proportions to establish a standard curve. Ten-microliter aliquots of serum containing different concentrations of the AFP-L3 antigens (12.5, 25, 50, 100, and 200 ng/ml) were added to the microarray, which was then incubated for 30 min at 37 °C (Fig. 5A,B). AFP-L3 was detected as described above.

The results were used to create a standard curve with the concentration of the AFP antigen on the x-axis and the OD value on the y-axis. The OD values on the y-axis and the corresponding concentration on the x-axis were read and multiplied by the dilution ratio to obtain the concentrations. Alternatively, the sample concentration could be calculated by determining the linear regression equation of the standard curve using the concentrations and OD values of the AFP antigen and then multiplying by the dilution ratio (Fig. 5B).

### ELISA

The serum AFP levels were quantified using the alpha-fetoprotein (AFP) human ELISA kit (Abcam ab108838) according to the manufacturer’s instructions. First, 50 μl of the alpha-fetoprotein standard or sample was added to each well. The wells were covered with sealing tape and incubated for two hours. Then, the wells were manually washed five times with 200 μl of 1X Wash Buffer. Second, 50 μl of 1X biotinylated alpha-fetoprotein antibody was added to each well and incubated for one hour. The microplate was washed as described above for the protein microarray. Third, 50 μl of 1X SP conjugate was added to each well and incubated for 30 min. The microplate was washed as described above for the protein microarray. Fourth, 50 μl of chromogen substrate was added to each well and incubated for 10 min or until the optimal blue color density was obtained. Finally, 50 μl of stop solution was added to each well. The color changed from blue to yellow. The absorbance was immediately determined on a microplate reader at a wavelength of 450 nm. The ELISA could be combined with chemiluminescence.

### Statistical analyses

Statistical analyses were conducted using SPSS version 17.0 (SPSS Inc., Chicago, IL, USA) for Windows. The results were compared using Pearson’s χ2-test. A P-value < 0.05 was considered statistically significant. The receiver operation characteristic (ROC) analysis was performed to determine the diagnostic performance of the protein microarray. The data were presented as the means ± SD (coefficient of variation) or medians (95% confidence interval [CI]).

## Additional Information

**How to cite this article**: Zhang, A. *et al*. A Chemiluminescent Protein Microarray Method for Determining the Seroglycoid Fucosylation Index. *Sci. Rep.* **6**, 31132; doi: 10.1038/srep31132 (2016).

## Supplementary Material

Supplementary Dataset 1

## Figures and Tables

**Table 1:** Protein microarray, Hotgen Biotech glycosyl capture spin column and ELISA analyses of the AFP and AFP-L3 levels in serum samples from patients with HCC and healthy controls.

Methods	Samples	AFP ≥ 20 ng/ml	0 < AFP < 20 ng/ml	AFP undetectable	AFP-L3% ≥10%	AFP-L3% <10%	AFP-L3 undetectable
Protein microarray	HCC (39)	35	2	2	22	4	9
	Healthy (39)	0	2	37	0	1	38
	Blank controls (10)	0	0	10	0	0	10
Biotech glycosyl capture spin column and ELISA	HCC (39)	34	3	2	21	5	9
	Healthy (39)	0	3	36	0	1	38
	Blank controls (10)	0	0	10	0	0	10

The values in the table represent the number of patients or healthy persons. There were no statistically significant differences between the results from the ELISA and protein microarrays in either group (P > 0.05; χ^2^-test). There was a statistically significant difference in the results from the protein microarray and ELISA between the HCC and healthy controls (P < 0.01).
