# Supplementary material for: A Chemiluminescent Protein Microarray Method for Determining the Seroglycoid Fucosylation Index
Source: Sci Rep. 2016 Aug 16;6:31132. doi: 10.1038/srep31132 (PMC4985809; doi:10.1038/srep31132)
Supplement: Supplementary Dataset 1 [file srep31132-s1.doc]

**Title**

A Chemiluminescent Protein Microarray Method for Determining the Seroglycoid Fucosylation Index

**Authors**

Aiying Zhang^1^, Sven Skog^2^, Shengqi Wang^3^, Yang Ke^4^, Yonghong Zhang^5^, Kang Li^5^, Ellen He^2^, and Ning Li*^1^

^1^Beijing Institute of Hepatology, Beijing YouAn Hospital, Capital Medical University, Beijing 100069, China. Aiying Zhang, e-mail: zhangaiying1996@163.com

^2^Sino-Swed Molecular Bio-Medicine Research Institute, Shenzhen 518057, China. Sven Skog, e-mail: svenisak@icloud.com; Ellen He, e-mail: ellen.he@sstkbiotech.com

^3^Department of Biotechnology, Beijing Institute of Radiation Medicine, Beijing 100850, China. Shengqi Wang, e-mail: wangshq1996@163.com

^4^Peking University Health Science Center, Beijing 100191, China. Yang Ke, e-mail: keyangbj@163.com

^5^Beijing YouAn Hospital, Capital Medical University, Beijing 100069, China.

Ning Li, e-mail: liningya@ccmu.edu.cn. Yonghong Zhang, e-mail: 13810108505@163.com. Kang Li, e-mail: [caaslk@163.com](mailto:caaslk@163.com);

*Corresponding Author

Prof. Ning Li,

YouAn Hospital, Beijing Institute of Hepatology, Capital Medical University

8 Xitoutiao, Youanmenwai, Fengtai District, Beijing 100069, China.

Phone: +86-10-83997001

Fax: +86-10-83997001

E-mail: liningya@ccmu.edu.cn

All microarray pictures

The following pictures show the scan chart of hepatic cancer and normal serum samples detected by AFP/lens culinaris lectin applied chips;

8 chips are used for detecting 39 hepatic cancer serum samples, 32 normal healthy serum samples and 9 blank controls.


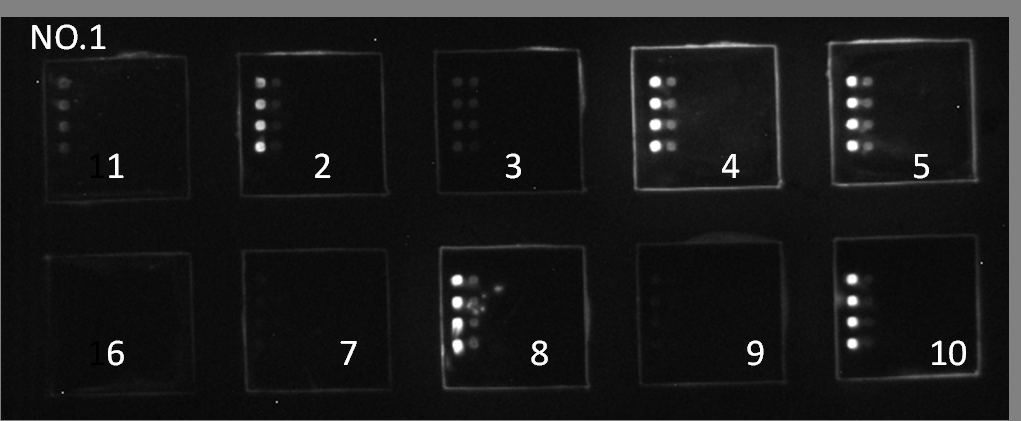


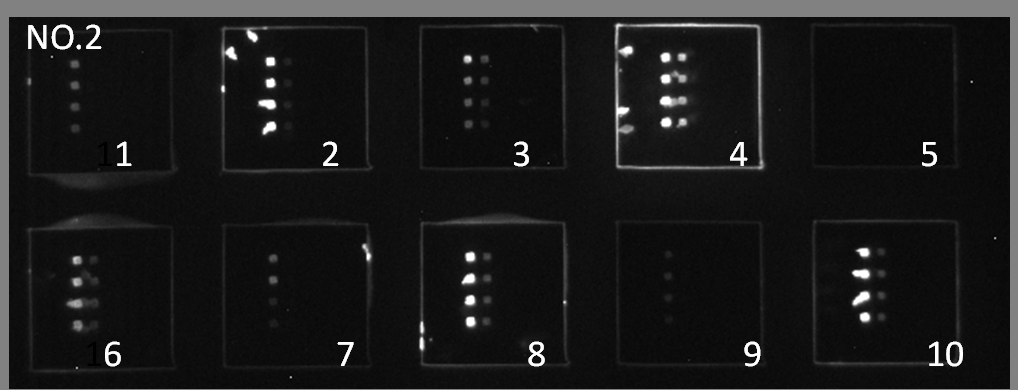


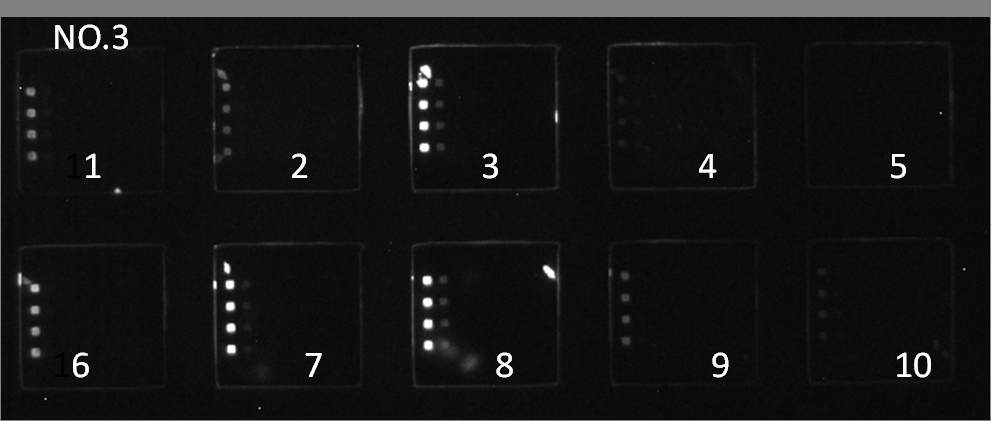


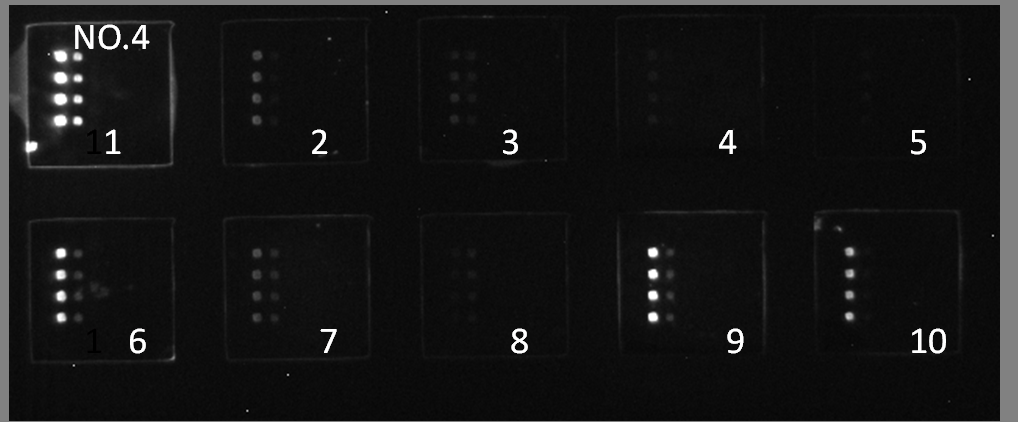


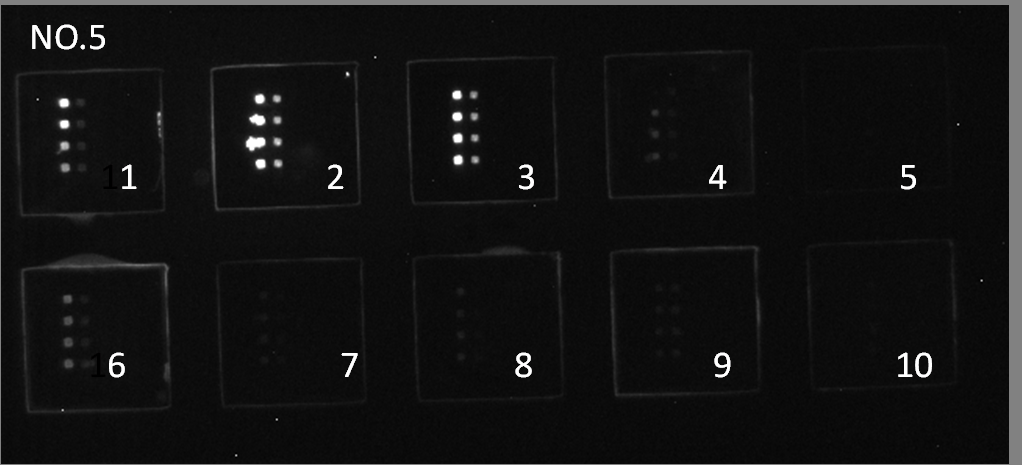


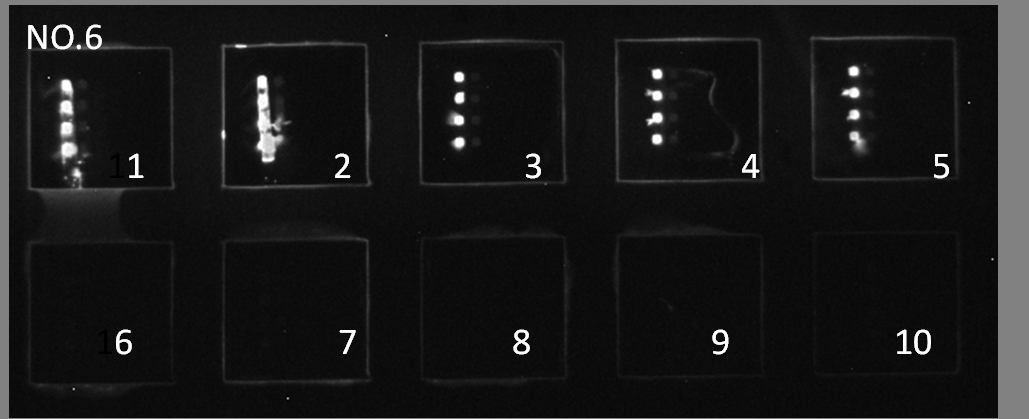


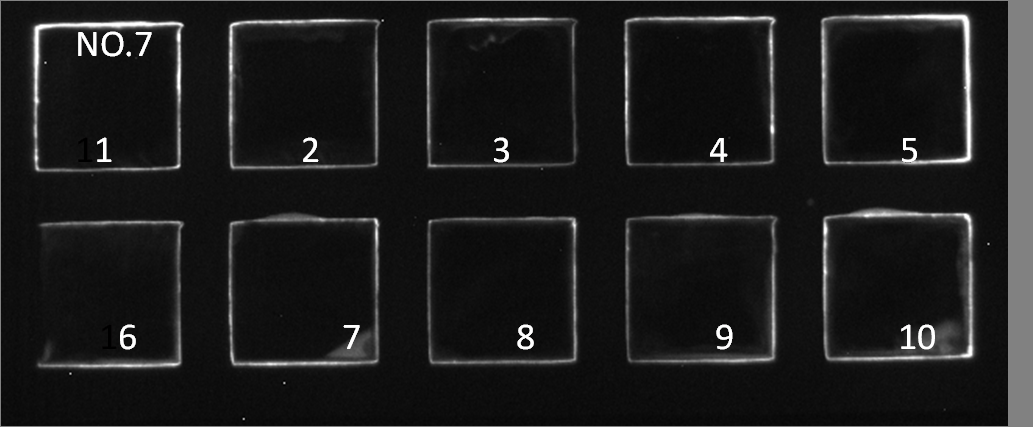


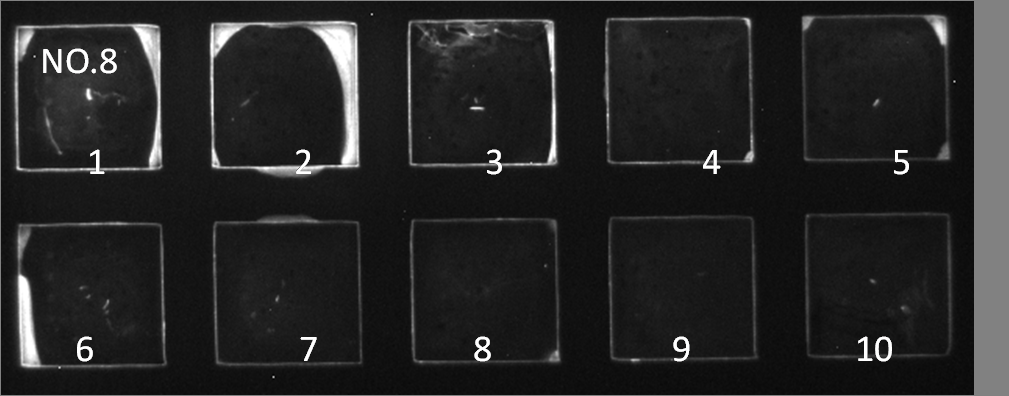


| **Table . Serum samples of HCC patients and healthy controls analyzed by**  **the chemiluminescent protein microarray.** | | | | | | | |  |  |
| --- | --- | --- | --- | --- | --- | --- | --- | --- | --- |
| No.Chip | Detection | Samples | AFP OD value | AFP-L3 OD value | AFP (ng/mL) | AFP-L3 (ng/mL) | AFP-L3/AFP | BCLC Staging | Treatment |
| No.1 | 1 | HCC | 55 | 20 | 11.65084 | — | — |  |  |
|  | 2 | HCC | 230 | 40 | 139.4814 | 27.16814 | 0.19478 | C | TACE+RFA |
|  | 3 | HCC | 80 | 30 | 29.91234 | 9.469027 | 0.316559 | B | TACE |
|  | 4 | HCC | 255 | 90 | 157.7429 | 115.6637 | 0.733242 | C | TACE |
|  | 5 | HCC | 255 | 100 | 157.7429 | 133.3628 | 0.845444 | B | / |
|  | 6 | C | 21 | 10 | — | — | — |  |  |
|  | 7 | N | 17 | 10 | — | — | — |  |  |
|  | 8 | HCC | 255 | 65 | 157.7429 | 71.41593 | 0.452736 | B |  |
|  | 9 | N | 20 | 18 | — | — | — |  |  |
|  | 10 | HCC | 245 | 18 | 150.4383 | — | — |  |  |
| No.2 | 1 | HCC | 90 | 12 | 37.21695 | — | — |  |  |
|  | 2 | HCC | 255 | 56 | 157.7429 | 55.48673 | 0.351754 | B | Resection |
|  | 3 | HCC | 150 | 49 | 81.04456 | 43.09735 | 0.531773 | C | TACE |
|  | 4 | HCC | 255 | 140 | 157.7429 | 204.1593 | 1.294254 | C | Resection |
|  | 5 | C | 10 | 10 | — | — | — |  |  |
|  | 6 | HCC | 160 | 50 | 88.34916 | 44.86726 | 0.50784 | C | TACE+RFA |
|  | 7 | HCC | 83 | 10 | 32.10373 | — | — |  |  |
|  | 8 | HCC | 255 | 70 | 157.7429 | 80.26549 | 0.508837 | B |  |
|  | 9 | N | 30 | 14 | — | — | — |  |  |
|  | 10 | HCC | 255 | 60 | 157.7429 | 62.56637 | 0.396635 | B | TACE |
| No.3 | 1 | HCC | 130 | 10 | 66.43535 | — | — |  |  |
|  | 2 | HCC | 80 | 10 | 29.91234 | — | — |  |  |
|  | 3 | HCC | 250 | 63 | 154.0906 | 67.87611 | 0.440495 | A | TACE |
|  | 4 | HCC | 25 | 10 | — | — | — |  |  |
|  | 5 | C | 10 | 10 | — | — | — |  |  |
|  | 6 | HCC | 160 | 10 | 88.34916 | — | — |  |  |
|  | 7 | HCC | 255 | 34 | 157.7429 | 16.54867 | 0.104909 | A | TACE+RFA |
|  | 8 | HCC | 255 | 60 | 157.7429 | 62.56637 | 0.396635 | B | Resection |
|  | 9 | HCC | 70 | 10 | 22.60774 | — | — |  |  |
|  | 10 | HCC | 50 | 10 | 7.998539 | — | — |  |  |
| No.4 | 1 | HCC | 255 | 230 | 157.7429 | 363.4513 | 2.304074 | B | / |
|  | 2 | HCC | 64 | 20 | 18.22498 | — | — |  |  |
|  | 3 | N | 20 | 20 | — | — | — |  |  |
|  | 4 | N | 20 | 10 | — | — | — |  |  |
|  | 5 | C | 10 | 10 | — | — | — |  |  |
|  | 6 | HCC | 255 | 70 | 157.7429 | 80.26549 | 0.508837 | B |  |
|  | 7 | HCC | 90 | 40 | 37.21695 | 27.16814 | 0.729994 | 0 | TACE |
|  | 8 | N | 20 | 20 | — | — | — |  |  |
|  | 9 | HCC | 255 | 70 | 157.7429 | 80.26549 | 0.508837 | 0 | TACE |
|  | 10 | HCC | 230 | 12 | 139.4814 | — | — |  |  |
| No.5 | 1 | HCC | 255 | 55 | 157.7429 | 53.71681 | 0.340534 | A | TACE+RFA |
|  | 2 | HCC | 255 | 197 | 157.7429 | 305.0442 | 1.933807 | 0 | TACE+RFA |
|  | 3 | HCC | 255 | 180 | 157.7429 | 274.9558 | 1.743063 | B |  |
|  | 4 | N | 40 | 20 | 0.693937 | — | — |  |  |
|  | 5 | C | 10 | 10 | — | — | — |  |  |
|  | 6 | HCC | 76 | 30 | 26.9905 | 9.469027 | 0.350828 | B | Resection |
|  | 7 | N | 20 | 13 | — | — | — |  |  |
|  | 8 | N | 30 | 10 | — | — | — |  |  |
|  | 9 | N | 20 | 20 | — | — | — |  |  |
|  | 10 | C | 10 | 10 | — | — | — |  |  |
| No.6 | 1 | HCC | 255 | 30 | 157.7429 | 9.469027 | 0.060028 | B |  |
|  | 2 | HCC | 255 | 27 | 157.7429 | 4.159292 | 0.026368 | A | Resection |
|  | 3 | HCC | 255 | 27 | 157.7429 | 4.159292 | 0.026368 | A | RFA |
|  | 4 | HCC | 255 | 30 | 157.7429 | 9.469027 | 0.060028 | C |  |
|  | 5 | HCC | 255 | 20 | 157.7429 | — | — |  |  |
|  | 6 | C | 10 | 10 | — | — | — |  |  |
|  | 7 | N | 10 | 10 | — | — | — |  |  |
|  | 8 | N | 10 | 10 | — | — | — |  |  |
|  | 9 | N | 10 | 10 | — | — | — |  |  |
|  | 10 | N | 10 | 10 | — | — | — |  |  |
| No.7 | 1 | N | 10 | 10 | — | — | — |  |  |
|  | 2 | N | 10 | 10 | — | — | — |  |  |
|  | 3 | N | 10 | 10 | — | — | — |  |  |
|  | 4 | N | 10 | 10 | — | — | — |  |  |
|  | 5 | C | 10 | 10 | — | — | — |  |  |
|  | 6 | N | 10 | 10 | — | — | — |  |  |
|  | 7 | N | 10 | 10 | — | — | — |  |  |
|  | 8 | N | 10 | 10 | — | — | — |  |  |
|  | 9 | N | 10 | 10 | — | — | — |  |  |
|  | 10 | N | 10 | 10 | — | — | — |  |  |
| No.8 | 1 | N | 10 | 10 | — | — | — |  |  |
|  | 2 | N | 10 | 10 | — | — | — |  |  |
|  | 3 | N | 10 | 10 | — | — | — |  |  |
|  | 4 | N | 10 | 10 | — | — | — |  |  |
|  | 5 | C | 10 | 10 | — | — | — |  |  |
|  | 6 | N | 10 | 10 | — | — | — |  |  |
|  | 7 | N | 10 | 10 | — | — | — |  |  |
|  | 8 | N | 10 | 10 | — | — | — |  |  |
|  | 9 | N | 10 | 10 | — | — | — |  |  |
|  | 10 | N | 10 | 10 | — | — | — |  |  |
|  | 1 | N | 39 | 20 | — | — | — |  |  |
|  | 2 | N | 20 | 15 | — | — | — |  |  |
|  | 3 | N | 30 | 20 | — | — | — |  |  |
|  | 4 | N | 48 | 25 | 6.537619 | 0.619469 | 0.094755 |  |  |
| N0. 9 | 5 | C | 10 | 10 | — | — | — |  |  |
|  | 6 | N | 28 | 15 | — | — | — |  |  |
|  | 7 | N | 12 | 10 | — | — | — |  |  |
|  | 8 | N | 10 | 10 | — | — | — |  |  |
|  | 9 | N | 13 | 10 | — | — | — |  |  |
|  | 10 | N | 10 | 10 | — | — | — |  |  |
|  | | | | | | | |  |  |

Abbreviations: HCC, HCC serum; N, normal, healthy human serum; C, blank; —, no signal; OD-value, chemiluminescent pixel;

0: very early stage HCC or stage 0; A: early stage HCC or stage A; B: stage B; C: stage C;

TACE: Transcatheter arterial chemoembolization; RFA: Radiofrequency ablation
